# Phylogeographic diversity and hybrid zone of *Hantaan orthohantavirus* collected in Gangwon Province, Republic of Korea

**DOI:** 10.1371/journal.pntd.0008714

**Published:** 2020-10-09

**Authors:** Geum-Young Lee, Won-Keun Kim, Kyungmin Park, Seung-Ho Lee, Jusun Hwang, Jin Sun No, Seungchan Cho, Daesang Lee, Dong-Hyun Song, Se Hun Gu, Man-Seong Park, Seong Tae Jeong, Young-Su Kim, Jin-Won Song

**Affiliations:** 1 Department of Microbiology, College of Medicine, Korea University, Seoul, Republic of Korea; 2 Department of Microbiology, College of Medicine, Hallym University, Chuncheon, Republic of Korea; 3 Institute of Medical Science, College of Medicine, Hallym University, Chuncheon, Republic of Korea; 4 Wildlife Ecology & Genomics Laboratory, College of Forest & Environmental Science, Kangwon National University, Chuncheon, Republic of Korea; 5 4th R&D Institute, Agency for Defense Development, Daejeon, Republic of Korea; 6 Infectious Disease Research Department, Gangwon Institute of Health and Environment, Chuncheon, Republic of Korea; Faculty of Science, Ain Shams University (ASU), EGYPT

## Abstract

**Background:**

*Hantaan orthohantavirus* (Hantaan virus, HTNV), harbored by *Apodemus agrarius* (the striped field mouse), causes hemorrhagic fever with renal syndrome (HFRS) in humans. Viral genome-based surveillance at new expansion sites to identify HFRS risks plays a critical role in tracking the infection source of orthohantavirus outbreak. In the Republic of Korea (ROK), most studies demonstrated the serological prevalence and genetic diversity of orthohantaviruses collected from HFRS patients or rodents in Gyeonggi Province. Gangwon Province is a HFRS-endemic area with a high incidence of patients and prevalence of infected rodents, ROK. However, the continued epidemiology and surveillance of orthohantavirus remain to be investigated.

**Methodology/Principal findings:**

Whole-genome sequencing of HTNV was accomplished in small mammals collected in Gangwon Province during 2015–2018 by multiplex PCR-based next-generation sequencing. To elucidate the geographic distribution and molecular diversity of viruses, we conducted phylogenetic analyses of HTNV tripartite genomes. We inferred the hybrid zone using cline analysis to estimate the geographic contact between two different HTNV lineages in the ROK. The graph incompatibility based reassortment finder performed reassortment analysis. A total of 12 HTNV genome sequences were completely obtained from *A*. *agrarius* newly collected in Gangwon Province. The phylogenetic and cline analyses demonstrated the genetic diversity and hybrid zone of HTNV in the ROK. Genetic exchange analysis suggested the possibility of reassortments in Cheorwon-gun, a highly HFRS-endemic area.

**Conclusions/Significance:**

The prevalence and distribution of HTNV in HFRS-endemic areas of Gangwon Province enhanced the phylogeographic map for orthohantavirus outbreak monitoring in ROK. This study revealed the hybrid zone reflecting the genetic diversity and evolutionary dynamics of HTNV circulating in Gangwon Province. The results arise awareness of rodent-borne orthohantavirus diseases for physicians in the endemic area of ROK.

## Author summary

The genetic and molecular epidemiological studies on small mammals derived from hemorrhagic fever with renal syndrome (HFRS)-endemic areas have consistently conducted for the public health surveillance and mitigation of orthohantavirus outbreak in the Republic of Korea (ROK). Implementing viral genome-based surveillance at new expansion sites that may identify HFRS risks is critical for tracking the location of orthohantavirus infections and diagnosing HFRS. In the present study, whole-genome sequences of Hantaan virus (HTNV) from small mammals in Gangwon Province were recovered using multiplex PCR-based next-generation sequencing during 2015–2018. In HFRS-endemic regions including Cheorwon-gun, Chuncheon-si, and Hwacheon-gun, additional HTNV genome sequences contribute to establish a high-resolution phylogeographic map for tracking emerging orthohantavirus-induced diseases. The cline analysis revealed a remarkable hybrid zone by showing spatial contact regions of HTNV at two sites (Cheorwon-gun and Hwacheon-gun) and the spatial separation and sequence divergence across genome segments of HTNV in Gangwon Province. These results demonstrate the genetic diversity and hybrid zone of HTNV circulating in Gangwon Province. These findings increase an awareness raising about HFRS in the endemic area of ROK.

## Introduction

Hantaan orthohantaviruses (Family *Hantaviridae*, Order *Bunyavirales*) are zoonotic negative-sense single-stranded RNA viruses containing large (L), medium (M), and small (S) segments [1]. The L segment encodes an RNA-dependent RNA polymerase (RdRp), the M segment encodes membrane glycoproteins (Gn and Gc), and the S segment contains a nucleocapsid (N) protein. Rodent-borne orthohantaviruses spread to rodents and humans through the inhalation of aerosols from infected animal excreta or rarely a bite. Orthohantavirus infections cause hemorrhagic fever with renal syndrome (HFRS) and hantavirus cardiopulmonary syndrome in humans [2]. HFRS is mainly caused by Old world orthohantaviruses, e.g., Hantaan virus (HTNV) carried by *Apodemus agrarius*; Seoul virus carried by *Rattus norvegicus* and *R*. *rattus*; Dobrava-Belgrade virus carried by *A*. *flavicollis*, *A*. *agrarius*, and *A*. *ponticus*; and Puumala virus (PUUV) carried by *Myodes glareolus* [3–5]. HFRS poses a critical public health threat with annual clinical cases of approximately 150,000–200,000 worldwide [6].

Phylogeographic analysis has become an essential tool for the public health surveillance and molecular epidemiology of infectious diseases when used for tracing the sources of epidemic infections [7]. Recently, the phylogenetic association between patients with HFRS and natural reservoirs demonstrated the putative infection location of HTNV [8]. Active surveillance in HFRS-endemic areas identified the infectious source of HTNV by real-time next-generation sequencing (NGS), epidemiological interview, and targeted rodent trapping [9]. Emerging orthohantavirus infections may occur at any time through contaminated urine, feces, or saliva in rodent-infested areas. To ascertain geographic prevalence and disease risk assessment of orthohantavirus in HFRS-endemic areas, ROK, genetic and molecular epidemiological studies on small mammals have consistently been conducted for decades [8,10–17]. Most studies have demonstrated the serological prevalence and genetic diversity of orthohantaviruses collected from HFRS patients or rodents in Gyeonggi Province [11–16]. In Gangwon Province, an administrative province in northeast ROK, approximately 371 HFRS cases have been reported from 2001–2019 [18]: Cheorwon-gun and Hwacheon-gun are highly HFRS-endemic areas. However, the continued surveillance and genetic study of HTNV remain to be investigated owing to the lack of virus genome sequences obtained from small mammals in Gangwon Province.

Hybrid zones are geographic regions in which two well-divergent taxa meet and mix [19]. The areas take various shapes from large scales of overlap to narrow contact regions or mosaic zones [20]. The particular zones are considered stable over evolutionary time [21]. The regions are characterized by variation in the genotype frequency across hybridization areas. A cline is formed by the frequencies of different genetic or phenotypic traits across hybrid regions. The contact zones continued by maintaining a balance between the homogenizing effect of dispersal and the diversifying effect of natural selection [22,23]. Hybrid zones were discovered in a variety of wild organisms [24–27]. Several studies have investigated the occurrence of hybrid zone between pathogens and their hosts in nature. The contact area plays a role in genetic diversity and speciation process in beak and feather disease viruses and their reservoirs [28]. Murine cytomegalovirus and their hosts formed a spatial contact in a hybrid zone maintained by natural selection [29]. Recently, a hybrid zone analysis revealed two distinct Tula virus (TULV) lineages were co-circulated in a geographical area in which two different evolutionary clades in the common vole (*Microtus arvalis*) interact and interbreed [30]. However, to the best of our knowledge, the hybrid zone of orthohantavirus in the ROK remains unknown.

In this study, epidemiological surveys of small mammals demonstrated the geographic prevalence of HTNV in Gangwon Province during 2015–2018. Targeted-enriched NGS elicited whole-genome sequences of HTNV acquired at the new expansion sites. The phylogeny of HTNV showed highly divergence and possible genetic exchanges of tripartite genomes in nature. In addition, the cline analysis suggested a hybrid zone of HTNV in Cheorwon-gun and Hwacheon-gun. This study provides significant insights into the phylogenetic diversity and evolutionary dynamics of orthohantaviruses in HFRS-endemic areas, ROK.

## Methods

### Ethics statement

All trappings of small mammals were carried out in accordance with the Ethical Guidelines of the Korea University Institutional Animal Care and Use Committee (KUIACUC #2016–49). Captured animals were euthanized via cardiac puncture under isoflurane anesthesia. To minimize hazards from potentially infected animals, workers wore thick rubber gloves and protective clothes. Collected rodents were placed in double plastic bags and transported to Korea University. Gloved hands were washed with a suitable disinfectant [31]. The experiment was conducted in the biosafety level 3 facility at Korea University.

### Sample collection

**Fig 1:**
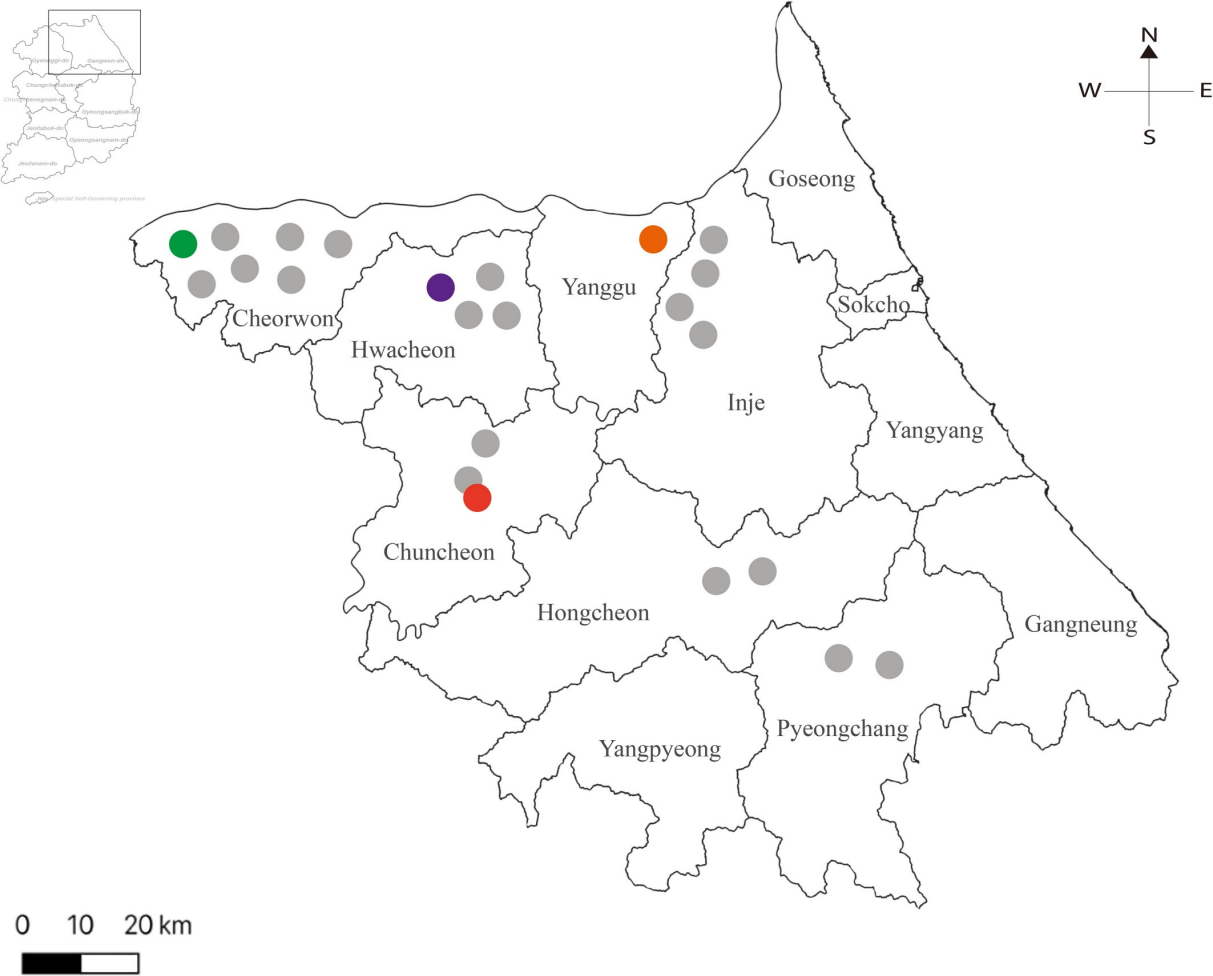
Geography of trapping sites of Hantaan virus (HTNV) collected in Gangwon Province, the Republic of Korea. The map shows different trapping sites where small mammals were captured in Gangwon Province from 2015 to 2018. The colored circle indicates HTNV RNA positive areas: Cheorwon-gun, green (Gwanu-ri); Chuncheon-si, red (Sinchon-ri); Hwacheon-gun, violet (Sanyang-ri); Yanggu-gun, orange (Mandae-ri). The grey circles represent the areas negative for HTNV specific RT-PCR: Cheorwon-gun (Jadeung-ri, Wasu-ri, Munhye-ri, Jigyeong-ri, Cheongyang-ri, and Gangpo-ri); Chuncheon-si (Geodu-ri and Cheonjeon-ri), Hongcheon-gun (Changchon-ri and Daehandong-gil); Hwacheon-gun (Daei-ri, Guman-ri, and Pungsan-ri); Inje-gun (Seohwa-ri, Deoksan-ri, Gaa-ri, and Cheondo-ri); Pyeongchang-gun (Nodong-ri and Ganpyeong-ri). Adobe Illustrator CS6 (http://www.adobe.com/products/illustrator.html) was used to create the map.

Small mammals were captured using Sherman live traps (8 by 9 by 23 cm; H. B. Sherman, Tallahassee, FL, USA) from tall grass and herbaceous vegetation habitats. Field trappings were performed in multiple sites, including Cheorwon-gun (gun = area) (Jadeung-ri (ri = village), Wasu-ri, Munhye-ri, Jigyeong-ri, Cheongyang-ri, Gangpo-ri, and Gwanu-ri), Hongcheon-gun (Changchon-ri and Daehandong-gil), Hwacheon-gun (Sanyang-ri, Daei-ri, Guman-ri, and Pungsan-ri), Inje-gun (Seohwa-ri, Deoksan-ri, Gaa-ri, and Cheondo-ri), Pyeongchang-gun (Nodong-ri and Ganpyeong-ri), and Yanggu-gun (Mandae-ri), in Gangwon Province from 2015 to 2018 **(Fig 1).** For each day, a total of 100 traps were set at intervals of 1–5 m and examined for 2–3 days. Traps for small mammals were sequentially numbered, placed in a secure container, and transported to Korea University where they were euthanized. In Chuncheon-si (si = city) (Geodu-ri, Sinchon-ri, and Cheonjeon-ri), small mammal trappings were performed in 20 sites, composed of four different habitats including grasslands, agricultural lands, ecotone area and forests. The geographic location was shown in the S1 Table. In total, 11 different species of 770 small mammals were collected. The captured animals consisted of 605 *A*. *agrarius*, 30 *A*. *peninsulae*, 61 *Crocidura lasiura*, 14 *C*. *shantungensis*, 5 *Micromys minutus*, 3 *Mus musculus*, 39 *M*. *regulus*, 2 *R*. *norvegicus*, 4 *Sorex mirabilis*, 2 *Tamias sibiricus*, and 5 *Tscherskia triton* **(Table 1)**. Traps positive for small mammals were sequentially identified by morphological characteristics [32]. Sera, lung, liver, spleen, and kidney tissues of the rodents and shrews were collected aseptically. Sera were isolated by centrifugation for 5 min at 4°C. Tissue samples were stored at -80°C until used.

**Table 1:** Summary for small mammals trapping in Gangwon Province from 2015 to 2018.

Species	Cheorwon-gun (%)	Chuncheon-si (%)	Hongcheon-gun (%)	Hwacheon-gun (%)	Inje-gun (%)	Pyeongchang-gun (%)	Yanggu-gun (%)	Total
*Apodemus agrarius*	93 (83.0)	124 (74.3)	61 (75.3)	124 (80.0)	153 (81.4)	21 (61.8)	29 (87.9)	605 (78.6)
*Apodemus peninsulae*	1 (0.9)	1 (0.6)	4 (4.9)	2 (1.3)	18 (9.6)	4 (11.8)	**-**^a^	30 (3.9)
*Crocidura lasiura*	4 (3.6)	26 (15.6)	4 (4.9)	12 (7.7)	12 (6.4)	3 (8.8)	**-**^a^	61 (7.9)
*Crocidura shantungensis*	1 (0.9)	9 (5.4)	1 (1.2)	2 (1.3)	-^a^	1 (2.9)	**-**^a^	14 (1.8)
*Micromys minutus*	2 (1.8)	2 (1.2)	-^a^	1 (0.6)	-^a^	-^a^	**-**^a^	5 (0.6)
*Mus musculus*	1 (0.9)	-^a^	-^a^	-^a^	-^a^	-^a^	2 (6.1)	3 (0.4)
*Myodes regulus*	7 (6.3)	3 (1.8)	7 (8.6)	12 (7.7)	5 (2.7)	4 (11.8)	1 (3.0)	39 (5.1)
*Rattus norvegicus*	1 (0.9)	1 (1.8)	-^a^	2 (1.3)	-^a^	-^a^	1 (3.0)	2 (0.3)
*Sorex mirabilis*	-^a^	-^a^	4 (4.9)	-^a^	-^a^	-^a^	**-**^a^	4 (0.5)
*Tamias sibiricus*	-^a^	1 (0.6)	-^a^	-^a^	-^a^	1 (2.9)	**-**^a^	2 (0.3)
*Tscherskia triton*	2 (1.8)	-^a^	-^a^	2 (1.3)	-^a^	-^a^	1 (3.0)	5 (0.6)
**Total**	**112**	**167**	**81**	**155**	**188**	**34**	**33**	**770**

^a^ No collection

### Mitochondrial DNA analysis

Total DNA was extracted from liver tissues using a High Pure PCR Template Preparation Kit (Roche, Basel, Switzerland). To verify a precise species of small mammals, the cytochrome *b* gene of mitochondrial DNA was amplified by universal primers; forward: 5’-CGA AGC TTG ATA TGA AAA ACC ATC GTT G-3’ and reverse: 5’-CTG GTT TAC AAG ACC AGA GTA AT’-3’ [33]. The HTNV-positive *A*. *agrarius* was phylogenetically confirmed by mitochondrial DNA cytochrome *b* gene.

### Indirect immunofluorescence antibody (IFA) test

Sera from *A*. *agrarius* were diluted 1:32 in phosphate buffered saline (PBS) [34]. The sera were added to wells of acetone-fixed Vero E6 cells infected with HTNV. The slides were incubated at 37°C for 30 min. After washing with PBS and distilled water (D.W.), fluorescein isothiocyanate-conjugated goat anti-mouse immunoglobulin G (IgG) antibody (ICN Pharmaceuticals, Laval, Quebec, Canada) was added. The plates were incubated at 37°C for 30 min and then washed with PBS and D.W. The pattern of virus-specific fluorescence was evaluated to be an indication of HTNV infection using a fluorescent microscope (Axioscope, Zeiss, Berlin, Germany).

### RNA extraction and reverse transcription-polymerase chain reaction (RT-PCR)

Total RNA was extracted from lung, liver, kidney, and spleen tissues using a FastPrep-24 5G Instrument (MP Biomedicals, USA) with TRI Reagent Solution (Ambion, Austin, Texas). Reverse transcription was conducted with 1 μg of total RNA using a High Capacity RNA-to-cDNA kit (Applied Biosystems, Foster City, CA, USA) with random hexamers and OSM55 (5′-TAG TAG TAG ACT CC-3′) [35].

HTNV-specific primer sequences were Han-L-F1 (outer): 5’-ATG TAY GTB AGT GCW GAT GC-3’, Han-L-R1 (outer): 5’-AAC CAD TCW GTY CCR TCA TC-3’, Han-L-F2 (inner): 5’-TGC WGA TGC HAC NAA RTG GTC-3’ and Han-L-R2 (inner): 5’-GCR TCR TCW GAR TGR TGD GCA A-3’ for the L segment [36]; G2F1 (outer): 5'-TGG GTG CAA GTG C-3’, G2-2 (outer): 5’ ACA TGC TGT ACA GCC TGT GCC-3’, G2-1 (inner): 5’-TGG GCT GCA AGT GCA TCA GAG-3’, G2-4 (inner): 5’-ATG GAT TAC AAC CCC AGC TCG-3’, OSS33 (outer): 5’-GAT ATG AAT GAT TGY TTT GT-3’, OSS34 (outer): 5’- CCA TCA GGG TCT YTC CA-3’, OSS35 (inner): 5’-TGT ATA ATT GGG ACW GAT TCT AA-3’ and OSS36 (inner): 5’-GCA AAG TTA CAT TTY TTC CT-3’ for the M segment [37,38]; HTN-S6 (outer): 5’-AGC TCI GGA TCC ATI TCA TC-3’, OSQ84 (outer): 5’-ATC TTA CAT CCT TTG TCG TCC C-3’, HTN-S4 (inner): 5’-GAI IGI TGT CCA CCA ACA TG-3’ and OSQ85 (inner): 5’-AGT TGT CCA CAG CCT CCT TT-3’ for the S segment [37,39]. First and second RT-PCR was performed at 94°C for 5 min, followed by 6 cycles of denaturation at 94°C for 30 s, annealing at 37°C for 40 s, and elongation at 72°C for 1 min; then, 32 cycles of denaturation at 94°C for 30 s, annealing at 42°C for 40 s, and elongation at 72°C for 1 min and a final cycle at 72°C for 5 min (ProFlex PCR System, Life Technology, CA, USA). PCR products were purified using a MinElute PCR purification kit (Qiagen, Hilden, Germany) or a QIAquick Gel Extraction Kit (Qiagen). Sequencing was performed in forward and reversed directions of each PCR product using a BigDye Terminator v3.1 Cycle Sequencing Kit (Applied Biosystems) on an automated sequencer (ABI 3730XL DNA Analyzer, Applied Biosystems). The whole-genome sequences of HTNV strains deposited in GenBank (Accession numbers: MT012546-MT012581).

### Real-time quantitative PCR (RT-qPCR)

RT-qPCR was performed at 95°C for 10 min, followed by 40 cycles at 95°C for 15 s and 60°C for 1 min using a Power SYBR Green PCR Master Mix (Applied Biosystems) on a QuantStudio 5 Real-Time PCR System (Applied Biosystems). The primer sequences of HTNV S segment included a forward primer (5’-TTA TTG TGC TCT TCA TGG TTG C-3’) and a reverse primer (5’-CAT CCC CTA AGT GGA AGT TGT C-3’) [40].

### Multiplex PCR-based next-generation sequencing (NGS)

cDNA was amplified using HTNV-specific primer mixtures and Solg 2X Uh-Taq PCR Smart mix (Solgent, Seoul, Republic of Korea) according to the manufacturer’s instruction. The enrichment was performed in 25 μL of reaction mixtures containing 12.5 μL of 2X Uh pre-mix, 2.0 μL of each primer mixture, 10.5 μL of D.W., and 1.0 μL of DNA template. Multiplex PCR was performed by a cycle at 95°C for 15 min, then 40 cycles and/or 25 cycles at 95°C for 20 s, 50°C for 40 s, 72°C for 1 min, and a cycle at 72°C for 3 min.

DNA libraries were prepared using a TruSeq Nano DNA LT sample preparation kit (Illumina, San Diego, USA) according to the manufacturer’s instructions. To obtain size-selected amplicons, cDNA templates were mechanically sheared using the M220 focused ultrasonicator (Covaris, Woburn, MA, USA). The cDNA amplicons were prepared by size-selection, A-tailing, and ligation with indexes and adaptors. The enrichment reaction contained 5 μL of PCR primer cocktail and 20 μL of enhanced PCR mixture. The quality of libraries was evaluated by a bioanalyzer using an Agilent DNA 1000 chip kit (Agilent Technologies, Santa Clara, USA). NGS was performed using MiSeq reagent V2 (Illumina) with 2×150 bp of MiSeq benchtop sequencer (Illumina).

### Rapid amplification cDNA ends (RACE) PCR

The 3’ and 5’ end sequences of viral genomes were acquired by rapid amplification cDNA end (RACE) PCR using a SMARTer RACE 5’/3’ Kit (Clontech Laboratories Inc., *Mountain View*, *CA*, *USA*) according to the manufacturer’s specifications. Both 3’ and 5’ ends of HTNV strains were empirically filled up by incomplete complementary sequences [41].

### Phylogenetic analysis

Whole-genome sequences of HTNV were aligned by the Clustal W algorithm (Lasergene program version 5, DNASTAR Inc. Madison, WI). The phylogenetic trees of HTNV were generated using the best-fit General Time Reversible (GTR) +gamma (G) +invariable (I) (for L and M segments) and T92+G (for S segment) models of evolution. Support for the topologies was assessed by bootstrapping for 1,000 iterations. Model optimizations were calculated for each data set, followed by the calculation of pairwise genetic distances between HTNV strains using MEGA 7.0 [42].

### Cline analysis

Geographic clines were inferred along one-dimensional transect axes crossing the contact areas for two diverged HTNV clades using the HZAR package [43]. The software includes functions for fitting molecular genetic or morphological data from hybrid zones to classic equilibrium cline models using the Metropolis–Hastings Markov chain Monte Carlo (MCMC) algorithm. HTNV strains were analyzed by performing 10^6^ generations of an MCMC sampling after 10^5^ burn-in iterations. Input files for cline analysis include information on locality distance, genotype frequency, and the number of samples. The hybridization frequency of trapping sites was displayed starting from the western-most locality in Gyeonggi Province. Genotype frequency data objects were created using the function hzar.doMolecularData1DPops in the software. The hzar.plot.obsData function plots data with mean frequencies for molecular clines and mean values for morphological clines.

### Genetic reassortment analysis

The graph incompatibility based reassortment finder (GiRaF) was performed to confirm reassortment events [44]. Nucleotide alignments of HTNV tripartite segments were used as an input source for MrBayes [45]. The best nucleotide substitution models were determined using MEGA 7.0. As input for the software, 1,000 unrooted candidate trees were estimated using the GTR+G+I substitution model, the burn-in 50,000 iterations (25%), and sampling every 200 iterations. These trees were used to simulate the phylogenetic uncertainty for segments; the parameters of the GiRaF were at the default settings. The default value of the confidence threshold was 0.7 for the data set; all events described using GiRaF were at over 0.9 confidence levels [46]. The process was repeated 10 times, with 10 independent MrBayes-based tree data per segment.

## Results

### Serological and molecular screening for HTNV

To detect anti-HTNV IgG, IFA test was performed using sera or heart fluid of *A*. *agrarius* captured in Gangwon Province. The representative HTNV-infected sample (Aa15-69) was shown by IFA test (S1 Fig). A total of 43/605 (7.1%) of *A*. *agrarius* samples were seropositive: 14/93 (15.1%) in Cheorwon-gun, 8/124 (6.5%) in Chuncheon-si, 2/61 (3.3%) in Hongcheon-gun, 10/124 (8.1%) in Hwacheon-gun, 3/153 (2.0%) in Inje-gun, and 6/29 (20.7%) in Yanggu-gun **(Table 2)**. Seropositivity of the rodents was not detected in Pyeongchang-gun. The seropositive samples were examined for viral RNA using HTNV-specific RT-PCR. In total, 26/43 (60.5%) seropositive of *A*. *agrarius* harbored HTNV RNA, consisting of 10/14 (71.4%) in Cheorwon-gun, 7/8 (87.5%) in Chuncheon-si, 4/10 (40.0%) in Hwacheon-gun, and 5/6 (83.3%) in Yanggu-gun.

**Table 2:** Serological and molecular screening results of Hantaan virus (HTNV) from *Apodemus agrarius* captured in Gangwon Province during 2015–2018.

Site	Year	Number of captured samples	Seropositivity for anti-HTNV IgG (%)	RT-PCR positivity (%)
Cheorwon-gun	2015	28	6/28 (21.4)	6/6 (100.0)
	2016	12	1/12 (8.3)	0/1
	2017	49	7/49 (14.3)	4/7 (57.1)
	2018	4	0/4	-^a^
	subtotal	93	14/93 (15.1)	10/14 (71.4)
Chuncheon-si	2017	100	7/100 (7.0)	7/7 (100.0)
	2018	24	1/24 (4.2)	0/1
	subtotal	124	8/124 (6.5)	7/8 (87.5)
Hongcheon-gun	2015	52	1/52 (1.9)	0/1
	2018	9	1/9 (11.1)	0/1
	subtotal	61	2/61 (3.3)	0/2
Hwacheon-gun	2015	27	5/27 (18.5)	4/5 (80.0)
	2016	42	1/42 (2.4)	0/1
	2017	55	4/55 (7.3)	0/4
	subtotal	124	10/124 (8.1)	4/10 (40.0)
Inje-gun	2015	109	3/109 (2.8)	0/3
	2016	44	0/44	-^a^
	subtotal	153	3/153 (2.0)	0/3
Pyeongchang-gun	2015	6	0/6	-^a^
	2018	15	0/15	-^a^
	subtotal	21	0/21	-^a^
Yanggu-gun	2018	29	6/29 (20.7)	5/6 (83.3)
	**Total**	**605**	**43/605 (7.1)**	**26/43 (60.5)**

^a^ Not-determined

### Multiplex PCR-based NGS of HTNV from *A*. *agrarius* captured in Gangwon Province

The coverage of genome sequences of 15 HTNV strains were 94.7–99.8% for L segments, 97.4–99.6% for M segments, and 99.2% for S segments **(Table 3)**. In total, whole-genome sequences of 12 HTNV strains were nearly obtained in Cheorwon-gun, Chuncheon-si, Hwacheon-gun, and Yanggu-gun. The whole-genome sequencing of Aa17-338 showed low coverages of the L and M segments corresponding to the lowest viral load. We acquired the 3’ and 5’ termini sequences of Aa17-421 using RACE PCR to complete the whole-genome sequencing of HTNV.

**Table 3:** Next-generation sequencing coverages of Hantaan virus (HTNV) from rodents collected during 2015 to 2018.

Site	Strain	Ct value	Origin	IFA titer	HTNV genome coverage (%)^b^		
					L segment	M segment	S segment
Sanyang-ri, Hwacheon-gun	Aa15-69^a^	27.2	Lung	1:2048	99.8	99.6	99.2
	Aa15-74^a^	21.7	Lung	1:2048	99.8	99.6	99.2
	Aa15-82^a^	28.9	Lung	1:128	99.8	99.6	99.2
	Aa15-84^a^	28.7	Lung	1:256	99.8	99.6	99.2
Gwanu-ri, Cheorwon-gun	Aa17-337^a^	28.4	Lung	1:2048	99.8	99.6	99.2
	Aa17-338	36.1	Spleen	1:64	42.4	54.2	99.2
	Aa17-353^a^	24.9	Lung	1:8192	99.8	99.6	99.2
Sinchon-ri, Chuncheon-si	Aa17-367^a^	25.0	Lung	1:1024	99.8	99.6	99.2
	Aa17-421^a^	23.6	Lung	1:8192	99.8	99.6	99.2
	Aa17-422^a^	20.1	Lung	1:4096	99.8	99.6	99.2
	Aa17-434	30.3	Spleen	1:512	94.7	97.6	99.2
Mandae-ri, Yanggu-gun	Aa18-164^a^	16.3	Lung	1:8192	99.8	99.6	99.2
	Aa18-177	27.7	Lung	1:16384	98.8	97.4	99.2
	Aa18-179^a^	27.1	Lung	1:16384	99.8	99.6	99.2
	Aa18-183	28.1	Spleen	1:8192	96.1	99.6	99.2
	Aa18-185^a^	20.5	Lung	1:4096	99.8	99.6	99.2

Aa, *Apodemus agrarius*; C_t_, cycle threshold; L, large; M, medium; S, small.

^a^ Whole-genome sequences of 12 HTNV strains were nearly recovered by multiplex PCR-based NGS.

^b^ Genome coverages were calculated by comparing genome sequences of the prototype HTNV 76–118 (L segment, 6,533 nt; M segment, 3,616 nt; S segment, 1,696 nt)

### Phylogenetic analysis

**Fig 2:**
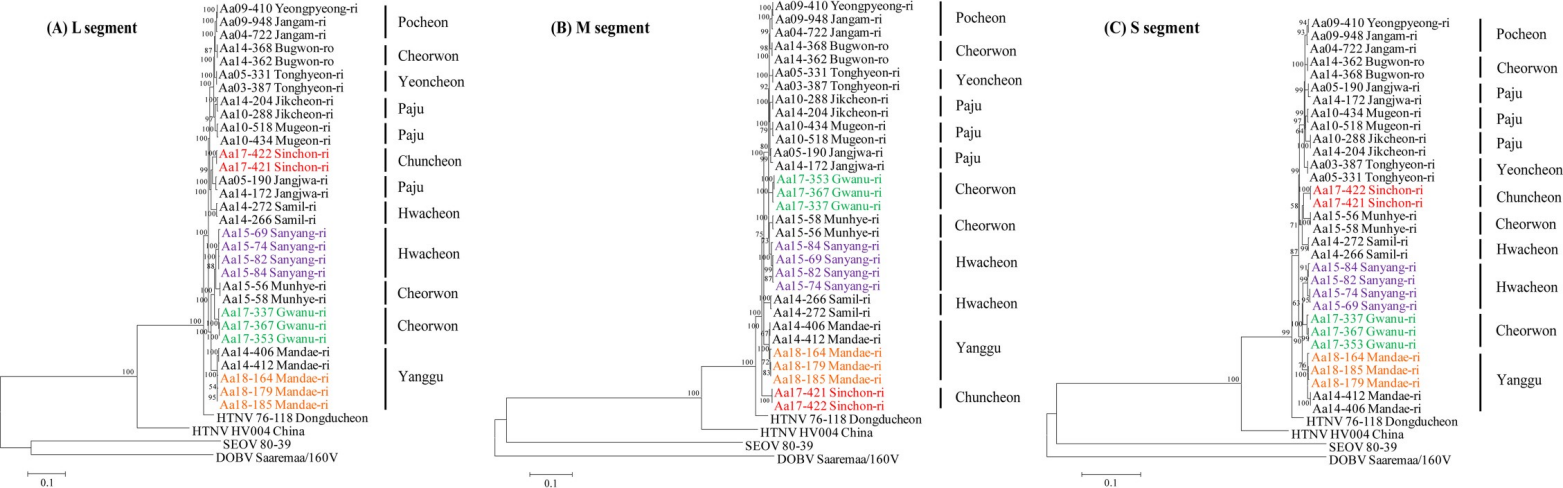
Phylogeographic analysis of Hantaan virus (HTNV) from rodents collected in Gangwon Province. Whole-genome sequences of HTNV from lung or spleen tissues of RT-PCR positive *A*. *agrarius* were obtained by multiplex PCR-based NGS and RACE PCR. Phylogenetic trees of HTNV (A) L segments, (B) M segments, and (C) S segments were generated by ML method. Branch lengths are proportional to the number of nucleotide substitutions, while vertical distances are for clarity. The numbers at each node are bootstrap probabilities, as determined for 1,000 iterations. The colors indicate specific sites in Gangwon Province; violet, Sanyang-ri in Hwacheon-gun; red, Sinchon-ri in Chuncheon-si; green, Gwanu-ri in Cheorwon-gun; orange, Mandae-ri in Yanggu-gun, respectively. The following HTNV sequences were used: Aa03-387 (L segment, KT934958; M segment, KT934992; S segment, KT935026), Aa04-722 (L segment, KU2071740; M segment, KU207182; S segment, KU207190), Aa05-190 (L segment, KT934959; M segment, KT934993; S segment, KT935027), Aa05-331 (L segment, KT934962; M segment, KT934996; S segment, KT935030), Aa09-410 (L segment, KU207177; M segment, KU207185; S segment, KU207193), Aa09-948 (L segment, KT934966; M segment, KT935000; S segment, KT935034), Aa10-288 (L segment, KT934969; M segment, KT935003; S segment, KT935037), Aa10-434 (L segment, KT934970; M segment, KT935004; S segment, KT935038), Aa10-518 (L segment, KT934971; M segment, KT935005; S segment, KT935039), Aa14-204 (L segment, KT934977; M segment, KT935011; S segment, KT935045), Aa14-406 (L segment, KT934985; M segment, KT935019; S segment, KT935053), Aa14-172 (L segment, KT934974; M segment, KT935008; S segment, KT935042), Aa14-362 (L segment, KT934981; M segment, KT935015; S segment, KT935049), Aa14-368 (L segment, KT934982; M segment, KT935016; S segment, KT935050), Aa14-412 (L segment, KT934987; M segment, KT935021; S segment, KT935055), Aa15-56 (L segment, KU207179; M segment, KU207187; S segment, KU207195), Aa15-58 (L segment, KU207180; M segment, KU207188; S segment, KU207196), HTNV 76–118 (L segment, NC005222; M segment, M14627; S segment, M14626), HTNV HV004 (L segment, JQ083393; M segment, JQ083394; S segment, JQ093395), DOBV Saaremaa/160V (L segment, AJ410618; M segment, AJ009774; S segment, AJ009773) and SEOV 80–39 (L segment, NC_005238; M segment, NC_005237; S segment, NC_005236).

The L and M segments of Aa15-69, Aa15-74, Aa15-82, and Aa15-84 in Hwacheon-gun (Sanyang-ri) phylogenetically grouped with HTNV in Cheorwon-gun (Munhye-ri) **(Fig 2)**. The HTNV S segment (Sanyang-ri) clustered with the HTNV in Cheorwon-gun (Gwanu-ri). The L segments of Aa17-421 and Aa17-422 in Chuncheon-si (Sinchon-ri) showed a genetic lineage with HTNV strains in Paju-si (Jangjwa-ri) and Hwacheon-gun (Samil-ri). The HTNV M segment (Sinchon-ri) formed a distinct group from all other strains in Gyeonggi and Gangwon Provinces. The HTNV S segment (Sinchon-ri) clustered with the HTNV in Cheorwon-gun (Munhye-ri) and Hwacheon-gun (Samil-ri). The L and M segments of Aa17-337, Aa17-353, and Aa17-367 in Cheorwon-gun (Gwanu-ri) clustered with the HTNV in Cheorwon-gun (Munhye-ri) and Hwacheon-gun (Sanyang-ri). The HTNV S segment (Gwanu-ri) phylogenetically grouped with HTNV in Hwacheon-gun (Sanyang-ri). The L segment of Aa18-164, Aa18-179, and Aa18-185 in Yanggu-gun (Mandae-ri) shared a common ancestor with HTNV in Cheorwon-gun (Munhye-ri and Gwanu-ri) and Hwacheon-gun (Sanyang-ri). The HTNV M segment (Mandae-ri) showed a genetic lineage with HTNV strains in Hwacheon-gun (Samil-ri). The HTNV S segment (Mandae-ri) formed a homologous genetic lineage with HTNV in Cheorwon-gun (Gwanu-ri) and Hwacheon-gun (Sanyang-ri).

### Hybrid zone analysis

**Fig 3:**
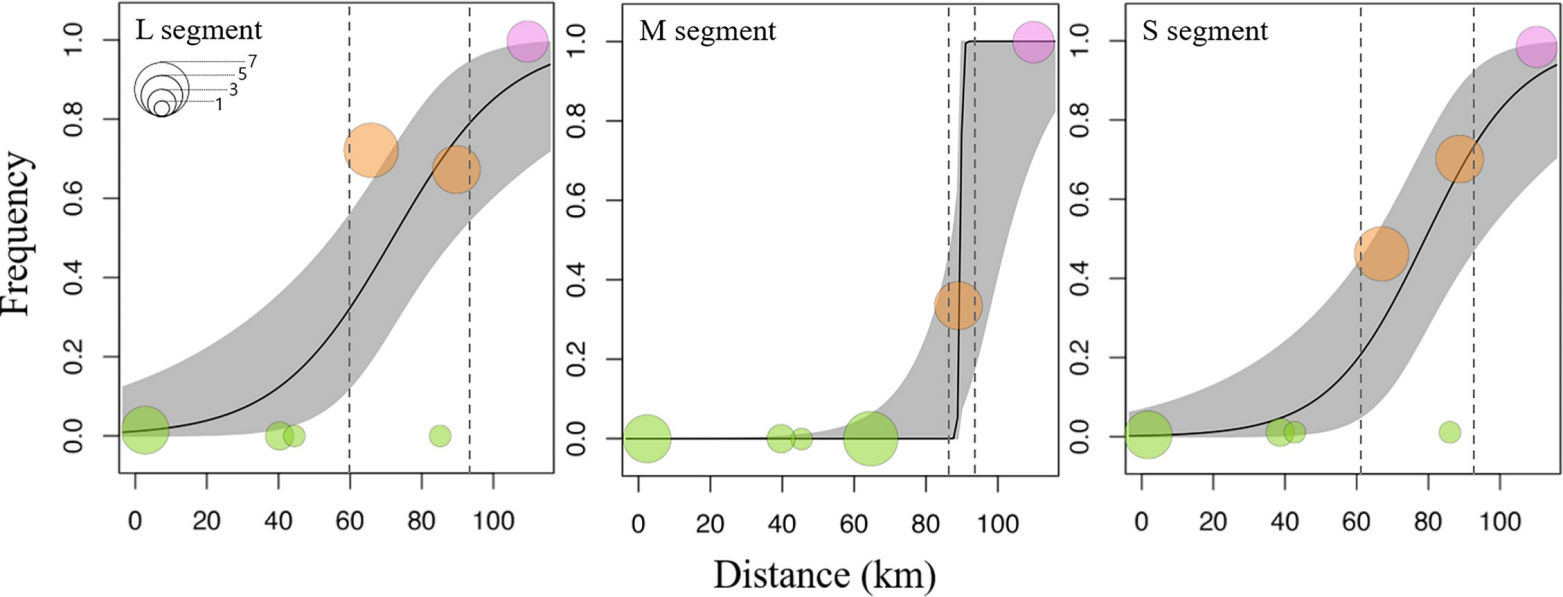
Geographic clines and phylogenetic clades representing the transition between genetic lineages of Hantaan virus (HTNV) in the Republic of Korea. Geographic clines show estimated changes in the population frequency of characters along geographic transects in Gyeonggi and Gangwon Provinces. The symbol sizes are equivalent to the number of samples and symbol colors to the genotype frequency per location (green, clade I; orange, hybrid zone; violet, clade II). The regions of 95% credible cline are shown in gray shade. Dotted lines indicate the distribution of the hybrid zone.

**Fig 4:**
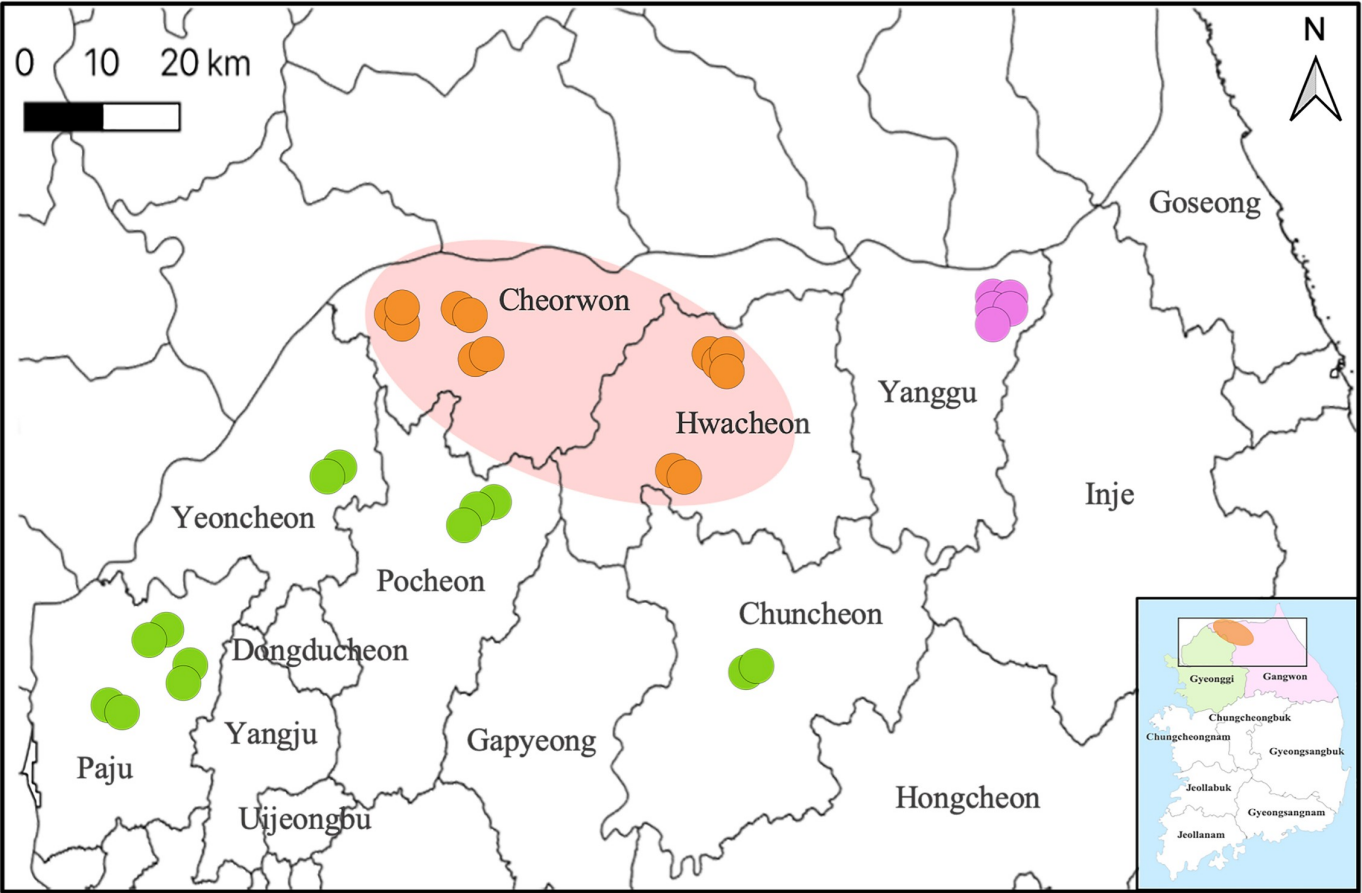
The hybrid zone of Hantaan virus (HTNV) found between two diverged clades in Gyeonggi and Gangwon Provinces. This map shows the geographic distribution of the genetic lineages of HTNV in Gyeonggi and Gangwon Provinces, Republic of Korea. The hybrid zone is observed at two sites (Cheorwon-gun and Hwacheon-gun) in Gangwon Province. The pink circle indicates the contact regions between two clades of HTNV in the hybrid zone. The colors represent specific genetic lineages: Green, clade I; orange, hybrid zone; violet, clade II. The geographic map was created by Quantum Geographical Information System (QGIS) software V.3.4.

A cline analysis inferred the change in the population frequency of diverged clades, defined by Gyeonggi and Gangwon Provinces, along geographic transects **(Table 4)**. The frequency variation was estimated according to each segment, and this analysis showed that the pattern of transition between HTNV clades in the L and S segments was spatially similar **(Fig 3)**. The transition pattern of the M segment was heterogeneous and significantly steeper compared to other segments. Geographic contacts were found between two phylogenetic lineages of HTNV in the ROK. In the L and S segments, hybrid zones were observed in Cheorwon-gun and Hwacheon-gun (64.3–89.4 km from Paju-si) whereas contact regions were detected only in Hwacheon-gun in the M segment. The range of the hybrid zone is approximately 20–25 km and distributed in Cheorwon-gun (Aa14-362, Aa14-368, Aa15-56, Aa15-58, Aa17-337, Aa17-353, and Aa17-367) and Hwacheon-gun (Aa14-266, Aa14-272, Aa15-69, Aa15-74, Aa15-82, and Aa15-84) **(Fig 4)**. Analysis of the genotype frequency for HTNV L, M, and S segments was divided into three clades; HTNV strains from Paju-si, Pocheon-si, Yeoncheon-gun, and Chuncheon-si showed the frequency of clade was *Ada*^*a*^ of 1 (Genotype of Gyeonggi) as referred to the Clade I. The clade frequency of HTNV strains from Yanggu-gun belonged to *Ada*^*b*^ of 1 (Genotype of Gangwon), as referred to the Clade II. Finally, Cheorwon-gun and Hwacheon-gun were selected as a hybrid zone between the clades I and II.

**Table 4:** Frequency of genotype clades of Hantaan virus and the number of samples for the hybrid zone between the Gyeonggi and Gangwon Provinces.

Clade	Site	Distance (km)	L segment		M segment		S segment		Number of samples
			*Ada*^*a*^	*Ada*^*b*^	*Ada*^*a*^	*Ada*^*b*^	*Ada*^*a*^	*Ada*^*b*^	
I	Paju-si	0	1	0	1	0	1	0	6
	Pocheon-si	39.8	1	0	1	0	1	0	3
	Yeoncheon-gun	45.3	1	0	1	0	1	0	2
	Chuncheon-si	84.8	1	0	-^a^	-^a^	1	0	2
Hybrid zone	Cheorwon-gun	64.3	0.286	0.714	1	0	0.571	0.429	7
	Hwacheon-gun	89.4	0.333	0.667	0.667	0.333	0.333	0.667	6
II	Yanggu-gun	112.4	0	1	0	1	0	1	5

*Ada*^*a*^; Frequency of clade I, *Ada*^*b*^; Frequency of clade II; L, large; M, medium; S, small.

^a^ Not-determined

### Genetic reassortment of HTNV

The occurrence of genetic exchange between segments (L and M, L and S, and M and S) was confirmed using the GiRaF program. The GiRaF analysis demonstrated that the reassortment event was detected between the M and S segments of HTNV in Aa15-56 and Aa15-58. The reassortment events were found in 100% of independent GiRaF runs by 10 times, and all events were supported over 0.9 confidence levels. However, additional genetic exchanges were not identified between L and the other two segments.

## Discussion

Epidemiological prevalence and genetic diversity of orthohantaviruses in nature reservoirs play a critical role in understanding of hantaviral diseases in HFRS-endemic areas. In 2005, HTNV genome sequences acquired from four HFRS patients at training sites near the Demilitarized Zone, ROK, showed an epidemiological link with viral sequences obtained by targeted rodent trapping at six training sites where patients had exercised [13]. These results help early diagnosis and prevention of HFRS patients by establishing a database to infer epidemiological and emergent dynamics of hantaviral genomes sequenced in endemic regions [47]. To intensify the resolution of phylogeographic map of orthohantaviruses, whole-genome sequences of HTNV were recovered from small mammals in Gangwon Province during 2015–2018. Additional genome sequences from three regions, Cheorwon-gun, Chuncheon-si, and Hwacheon-gun in Gangwon Province, were obtained to enhance the geographical data available for surveilling HFRS incidence by genomic epidemiology **(Fig 2)**. Here, HTNV strains were first detected in Chuncheon-si. Thus, there should be vigilance for potential human orthohantavirus infections in the region.

Hybrid zones are geographic regions in which genetically divergent populations contact and mix [23]. The areas allow to observe interactions with two different genotypes and the process of speciation [21,48]. The two diverged clades of PUUV were circulated in a single host lineage, and inter-lineage reassortments of PUUV were detected in northern Finland [26]. The hybrid zone of TULV demonstrates correlation between the distribution of two phylogenetic lineages in TULV and their host clades in the European common vole (*M*. *arvalis*) [30]. The spatial transition between TULV lineages was narrower than clades of their reservoirs. In this study, spatial contact regions of HTNV were investigated through the cline analysis. The results revealed a remarkable hybrid zone at two sites (Cheorwon-gun and Hwacheon-gun) in Gangwon Province and showed the spatial separation and sequence divergence across genome segments of HTNV **(Fig 3)**. Although the impact of fitness differences between individuals in the local population cannot be ignored, one might expect the hybrid zone to move in favor of the fitter genotype [49–52]. The genotype frequency (*Ada*^*a*^ of 1) of the M segment in Cheorwon-gun indicated the M segment is completely compatible with the clade I. In Hwacheon-gun, the HTNV M segment showed a selected genetic phenotype of the clade I with the *Ada*^*a*^ of 0.667 and *Ada*^*b*^ of 0.333. These results demonstrated that the M segment of HTNV may be preferentially compatible with the clade I in the hybrid zone. The genetic lineage of HTNV L segment in Cheorwon-gun showed higher frequency of *Ada*^*b*^ (Genotype of Gangwon) compared with the M and S segments. The results showed that HTNV L segment may be compatible with the L segment depending on the geographic origin.

Segmented RNA viruses have the capacity to exchange genome segments by genetic reassortment [53]. Several orthohantaviruses, including the PUUV, Sin Nombre virus (SNV), and HTNV, have been described with the intra- and inter-lineage reassortment between closely related variants [26,54–56]. In the previous study, reassortment analysis reported that a genetic exchange in HTNV might occur in nature [18]. The genetic exchange of SNV was reported on the M segment in nature and in vitro [57,58]. Andes virus also has high level molecular diversity in the M segment, resulting in five diverged clades related to geographic origins in the South Americas [59]. These reports consistently showed that the possible exchanges of the M segment had lower genetic compatibility requirements and higher genetic tolerance than the L and S segments [49,60,61]. However, a homologous association between the L and M segments was observed in PUUV [62]. Genetic reassortments of HTNV strains (2/11; 18.2%) in the hybrid zone was detected using GiRaF analysis. The GiRaF analysis demonstrated that Aa15-56 and Aa15-58 from Cheorwon-gun were reassortants that have phylogenetic heterogeneity between the M and S segments. However, additional reassortants were not found between the L and the other two segments. The genetic compatibility and homologous relationships among hantaviral genome segments remain to be studied.

This study led us the hypothesis that genetic exchange rates of HTNV may be associated with the hybrid zone. Co-circulation of different lineages within hybrid zones presented the opportunity to confer genetic exchanges and dominance of the virus genome between these two genetic groups. The genetic exchanges confer viral characteristics including fitness, transmission, and pathogenesis [63]. The genetic reassortments of different segments promoted the evasion of host immunity and the occurrence of epidemics in influenza A virus and rotavirus A [64–66]. Continued reassortment of human immunodeficiency virus gave rise to genetic variants with transmissibility and altered virulence in humans [67]. A genetic exchange of the M segment of DOBV occurred between low pathogenic DOBV-Aa and highly pathogenic DOBV-Af [68]. In this study, HTNV in Cheorwon-gun showed genome organization compatible with reassortments between Gyeonggi and Gangwon Provinces. The cline analysis demonstrated that Cheorwon-gun and Hwacheon-gun were a hybrid zone among HTNV population in northern areas, ROK. However, some questions remain to be further investigated: 1) whether the genetic exchange of hantaviruses associates with the co-existence of different genetic lineages; 2) whether the biological consequence of the reassortment affects the pathogenicity in humans.

In summary, the prevalence and distribution of HTNV in HFRS-endemic areas of Gangwon Province enhance the phylogeographic maps for the monitoring and response to orthohantavirus outbreaks in the ROK. The hybrid zone analysis reveals hybridization of HTNV strains and the reassortment analysis suggests a natural occurrence of HTNV genetic exchange in the ROK. The finding provides significant insights into the genetic diversity and evolutionary dynamics of HTNV circulating in Gangwon Province. This report increases awareness of the rodent borne-orthohantavirus disease for physicians in the endemic area of ROK.

## Supporting information

S1 Table Trapping sites of small mammals colleted in Gangwon Province. (DOCX)

S1 Fig Immunofluorescence on Hantaan virus (HTNV)-infected Vero E6 cells. (A) The representative HTNV-infected sample (Aa15-69) was shown in the S1 Fig. (B) The negative control was confirmed by PBS. (C) The positive control was an image of Vero E6 cells infected with HTNV 76–118 (the prototype of orthohantavirus). The arrow (red) is the antigen spot identified in the positive sample.
(TIF)
