# Supplementary material for: Phylogeographic diversity and hybrid zone of Hantaan orthohantavirus collected in Gangwon Province, Republic of Korea
Source: PLoS Negl Trop Dis. 2020 Oct 9;14(10):e0008714. doi: 10.1371/journal.pntd.0008714 (PMC7588125; doi:10.1371/journal.pntd.0008714)
Supplement: S1 Table — (DOCX) [file pntd.0008714.s001.docx]

**S1 Table.** **Trapping sites of captured small mammals** **in Gangwon Province.**

| **Trapping sites** | | **GPS coordinates** |
| --- | --- | --- |
| Cheorwon-gun | Jadeung-ri | 38°12′42.8′′N 127°25′29.1′′E |
|  | Wasu-ri | 38°12′59.6′′N 127°25′29′′E |
|  | Munhye-ri | 38°12′55.3′′N 127°20′36′′E |
|  | Jigyeong-ri | 38°12′26.8′′N 127°20′30.8′′E |
|  | Cheongyang-ri | 38°14′04.12′′N 127°21′28.92′′E |
|  | Gangpo-ri | 38°7′1′′N 127°17′45′′E |
|  | Gwanu-ri | 38°14′30′′N 127°13′10′′E |
| Chuncheon-si | Geodu-ri | 37°51′37′′N 127°46′15′′E |
|  | Sinchon-ri | 37°51′7′′N 127°47′36′′E |
|  | Cheonjeon-ri | 37°56′34′′N 127°46′34′′E |
| Hongcheon-gun | Changchon-ri | 37°45′34′′N 128°24′57′′E |
|  | Daehandong-gil | 37°45′29′′N 128°25′07′′E |
| Hwacheon-gun | Sanyang-ri | 38°13′56′′N 127°40′4.4′′E |
|  | Daei-ri | 38°6′10′′N 127°45′40′′E |
|  | Guman-ri | 38°4′58′′N 127°45′18′′E |
|  | Pungsan-ri | 38°9′54′′N 127°44′39′′E |
| Inje-gun | Seohwa-ri | 38°14′35.87′′N 128°13′9.10′′E |
|  | Deoksan-ri | 38°5′53′′N 128°11′23′′E |
|  | Gaa-ri | 38°8′20′′N 128°8′39′′E |
|  | Cheondo-ri | 38°13′19′′N 128°12′10′′E |
| Pyeonchang-gun | Nodong-ri | 37°41′19′′N 128°28′14E |
|  | Ganpyeong-ri | 37°40′6.4′′N 128°35′20E |
| Yanggu-gun | Mandae-ri | 38°16′7′′N 128°8′52′′E |
