# Supplementary figures and images for: Phylogeographic diversity and hybrid zone of Hantaan orthohantavirus collected in Gangwon Province, Republic of Korea
Source: PLoS Negl Trop Dis. 2020 Oct 9;14(10):e0008714. doi: 10.1371/journal.pntd.0008714 (PMC7588125; doi:10.1371/journal.pntd.0008714)

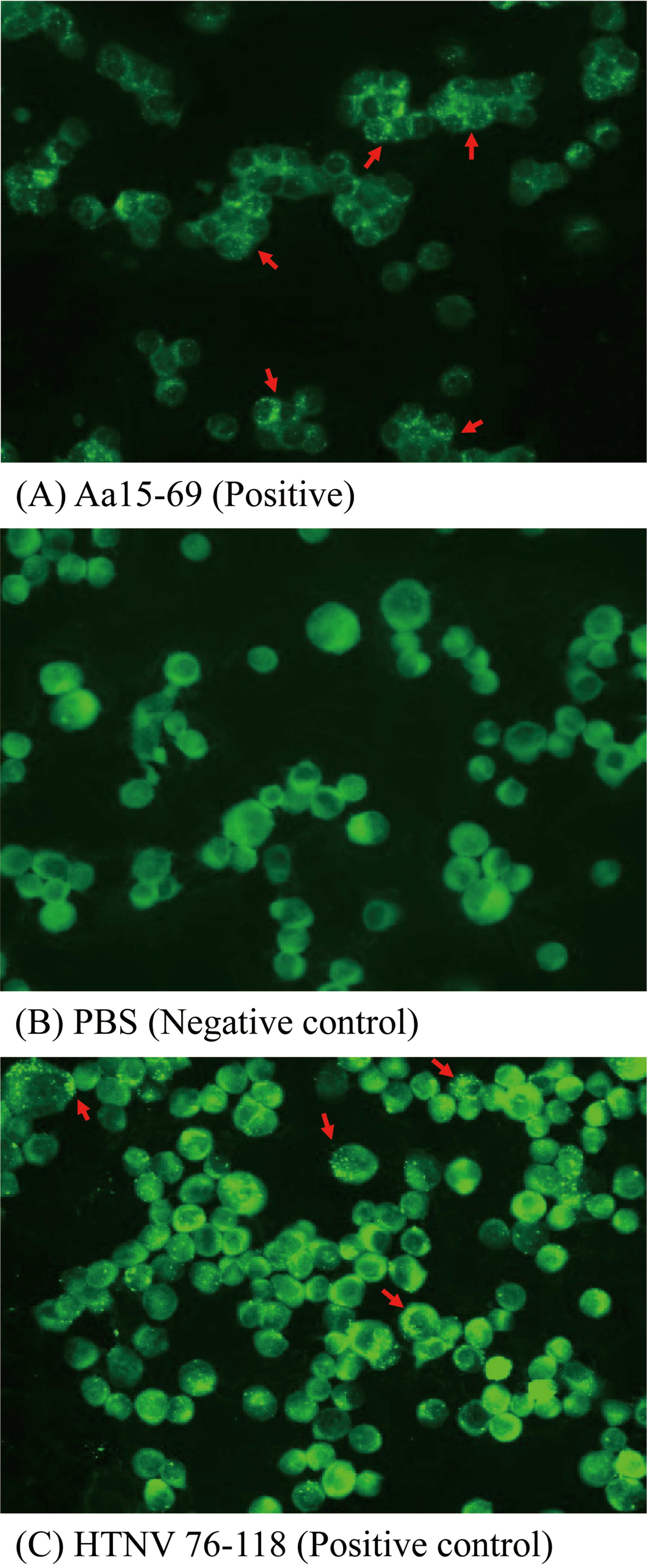

Supplement: S1 Fig — (A) The representative HTNV-infected sample (Aa15-69) was shown in the S1 Fig. (B) The negative control was confirmed by PBS. (C) The positive control was an image of Vero E6 cells infected with HTNV 76–118 (the prototype of orthohantavirus). The arrow (red) is the antigen spot identified in the positive sample. (TIF) [file pntd.0008714.s002.tif]
